# The Relationship Between Family Support and e-Learning Engagement in College Students: The Mediating Role of e-Learning Normative Consciousness and Behaviors and Self-Efficacy

**DOI:** 10.3389/fpsyg.2021.573779

**Published:** 2021-02-04

**Authors:** Hong Gao, Yangli Ou, Zhiyuan Zhang, Menghui Ni, Xinlian Zhou, Li Liao

**Affiliations:** ^1^School of Nursing, University of South China, Hengyang, China; ^2^Emergency Department, The Second Hospital University of South China, Hengyang, China

**Keywords:** e-learning engagement, family support, e-learning normative consciousness and behaviors, self-efficacy, college students

## Abstract

Due to the current COVID-19 pandemic, colleges and universities have implemented network teaching. E-learning engagement is the most important concern of educators and parents because this will directly affect student academic performance. Hence, this study focuses on students’ perceived family support and their e-learning engagement and analyzes the effects of e-learning normative consciousness and behaviors and self-efficacy on the relationship between family support and e-learning engagement in college students. Prior to this study, the relationship between these variables was unknown. Four structural equation models revealed the multiple mediating roles of e-learning normative consciousness and behaviors and self-efficacy in the relationship between family support and e-learning engagement. A total of 1,317 college students (mean age=19.51; 52.2% freshman) voluntarily participated in our study. The results showed that e-learning normative consciousness and behaviors and self-efficacy played significant and mediating roles between students’ perceived family support and e-learning engagement. Specifically, these two individual variables fully mediated the relationship between students’ perceived family support and e-learning engagement. The multiple mediation model showed that family members can increase family support of their children by creating a household environment conducive to learning, displaying positive emotions, demonstrating the capability to assist their children, advocating the significance of learning normative consciousness and behaviors, and encouraging dedicated and efficient learning. The findings complement and extend the understanding of factors influencing student e-learning engagement.

## Introduction

The theme of this paper belongs to the category of psychological oriented educational intervention, and its research history can be traced back to the early 20th century. Under the diverse theoretical orientations, educational interventions are to explore a variety of simple to very complex interventions, including a variety of measurements and methods, in order to solve the decidedly pragmatic problems in the learning process (Pressley et al., 2006). Psychological oriented educational interventions are invariably inspired by theories of development, motivation, cognition, or learning (Pressley et al., 2006). At present, many educational interventions measurements and methods are less popular. However, educational intervention research plays a fundamental and decisive role in the reformation of teaching. Many research-validated interventions are adopted by educators in priority (Buckley et al., 2010; Pickering et al., 2018).

The Internet has become an indispensable part of our daily life and has also become the greatest source of information and knowledge worldwide. Web-based learning is an accessible and effective learning method that educators and students use to supplement or replace traditional learning, especially during the outbreak of the COVID-19. In recent years, a large number of studies have reported that e-learning has been widely used in various disciplines, such as clinical medical education (Aloia and Vaporciyan, 2019; Grabowski et al., 2020; Tretter et al., 2020), nursing education (Sinclair et al., 2016; Voutilainen et al., 2017; Kim and Park, 2019), and higher education (Chu et al., 2019; He and Yusop, 2020; Zhang et al., 2020). Therefore, the advantages of e-learning, such as flexibility in learning, convenience in enabling learners to review subjects, and more vivid and authentic displays of cases, are also recognized by educators and learners (Fagö-Olsen et al., 2020). However, following the popularization of online teaching, many educators have begun to worry about the effects on student learning. Hence, this study focuses on family support and e-learning engagement and analyzes the possible factors influencing student e-learning engagement.

### Learning Engagement

Student learning engagement is an important factor affecting the learning effect, especially in the network learning environment, which lacks teacher supervision. At present, due to the COVID-19 pandemic, colleges and universities have implemented network teaching. From the perspective of positive psychology, student learning engagement has received unprecedented attention (Yan et al., 2018). Learning engagement consists of absorption, dedication, and vigor, which is a positive mental state in learning-related activities (Ouweneel et al., 2011). The absorption dimension can describe a state of learning in which students are fully engaged in learning and experience the joy of learning. The dedication dimension can describe students’ sense of pride and meaning, their tremendous enthusiasm to learn, their wholehearted devotion to their studies and their courage to accept any challenges. The vigor dimension can describe a physical condition in which learners are full of energy as a result of learning, study hard without fatigue, and persevere in the face of difficulties (Siang and Santoso, 2016). The higher degree of student learning engagement can be a reflection of these learners having a greater sense of self-control, mastering better learning strategies, and having higher levels of physical and mental health (Wefald and Downey, 2009).

### Family Support and Learning Engagement

Many external factors can influence learning engagement, including the external environment, school support, and family support (Garciareid et al., 2015; Mazurenko and Hearld, 2015; Kelly and Zhang, 2016). Due to the impact of the COVID-19 pandemic, college students in China participate in online courses at home. In this case, the influence of family support on student e-learning engagement is more obvious. Family support refers to environmental support, emotional support, and capability support. In the process of students developing learning potential, their interactions with their proximal social environment (e.g., family environment) are of utmost importance (Mudrak et al., 2019; Mudrák et al., 2020). Students’ family support, including family socioeconomic status, parental support, parental expectations, family social and material resources, etc., affects the development of learning competencies and learning motivation (Elliot et al., 2017; Ericsson et al., 2018). Therefore, it is essential to more fully investigate the relationship between family support and e-learning engagement and to identify the contributions of different factors, such as learning normative consciousness and behaviors and self-efficacy, to this association.

### Learning Normative Consciousness and Behaviors as a Mediator

Many internal factors can influence learning engagement, including needs, motivation, and personality traits (e.g., learning normative consciousness and learning behaviors; Martin et al., 2016; Yamada et al., 2017; Cilliers et al., 2018). The normative consciousness of learning is an intuitive confidence that students deliberately form in the process of learning. Learning normative consciousness is closely associated with the autonomy concept, particularly in regards to learning behavior, motivation, and metacognition, which enables students to take responsibility for their own learning (Schunk and Zimmerman, 1998). Greene et al. (2010) indicated that students who do well in the network-based learning environment can manage their e-learning by forming learning normative consciousness on the basis of metacognitive and cognitive processes, such as student self-monitoring, setting suitable learning objectives, and ensuring the effectiveness of their learning strategies. According to converging theories, learning normative consciousness and behaviors are the crystallization of the experience accumulated by human long-term learning. Converging studies in machine learning, biology, and neuroscience suggests that students actively acquire information in the learning environment, a process which is greatly influenced by learning normative consciousness and behaviors (Gottlieb and Oudeyer, 2018; Yang et al., 2018). Meanwhile, students’ learning normative consciousness and behaviors are influenced by their learning environment, such as family support. In rich and complex learning environments, learning engagement is likely to be greatly reduced, or uncertainly reduced, while learning normative consciousness and behaviors can help avoid bad guidance and sub-optimal learning engagement (Tschantz et al., 2020). Therefore, on the basis of the above studies, we hypothesized that learning normative consciousness and behaviors as mediators affect the relationship between family support and student e-learning engagement.

### Student e-Learning Self-Efficacy as a Mediator

Self-efficacy is the product of the development of social cognitive theory (Bandura, 1994). Some researchers believe that self-efficacy refers to the confidence of a learner to successfully complete a task, which can be used as a measure of confidence (Bandura, 1986; Salles, 2017). Students’ perceived self-efficacy is a subjective assessment of their capacity to achieve certain personal goals and to overcome difficulties, which can improve students’ learning engagement and ultimately achieve good academic achievements (Bandura, 1997; Cook and Artino, 2016; Reichwein Zientek et al., 2017). However, psychologists think self-efficacy is malleable and will increase or decrease with the influence of environmental factors (Klassen, 2004; Pekrun, 2006).

Forming good learning normative consciousness and behaviors in the long-term learning process can improve student learning self-efficacy, and this self-efficacy will be relatively stable. In e-learning environments, the roles of student e-learning self-efficacy and learning normative consciousness and behaviors in the mechanisms that govern how family support affects their learning engagement are still unclear.

The aim of this study was to determine the potential mechanisms underlying the relationship between family support and student e-learning engagement. Specifically, we will examine how family support influences student e-learning engagement through their e-learning normative consciousness and behaviors and e-learning self-efficacy.

### Hypotheses of This Study

In our study, multiple mediation models were predicted to examine the roles of student e-learning self-efficacy and e-learning normative consciousness and behaviors in the mechanisms that govern how family support affects their e-learning engagement. Specifically, we proposed the following hypotheses:

Hypothesis 1: Family support of e-learning is directly associated with student e-learning engagement.
Hypothesis 2: Student e-learning normative consciousness and behaviors and self-efficacy will mediate the association between family support and student e-learning engagement.

## Materials and Methods

### Participants

The participants of our study were 1,317 college students from a comprehensive research university in Hengyang, Hunan Province, China, selected using convenience sampling. All represented class subjects included freshman, sophomore, and junior and senior students, among which liberal arts and sciences accounted for 34.9 and 65.1%, respectively. All of the subjects were investigated after 60days of online learning. Students’ online learning methods mainly include participating in live broadcast and watching teaching videos. College students from the selected classes who provided their informed consent were invited to complete questionnaires, which also included demographic information, such as age, grade level, and parents’ education level. We chose a professional platform what is named “Wenjuanxing” for questionnaire survey. From April 13 to April 26, 2020, a total of 1,500 students were enrolled, and a total of 1,317 valid questionnaires were collected, indicating an efficiency of 87.8%. The mean age was 19.51±1.51years. The mean values of the family support score (14–98), learning normative consciousness and behaviors score (6–42), learning self-efficacy score (18–126), and student learning engagement score (16–112) of respondents were 80.45, 32.93, 88.49, and 74.18, respectively. Furthermore, the freshman, sophomore, junior, and senior ratios of the respondents were 52.2, 27.6, 10.2, and 10.0%, respectively. More than 70% of respondents’ parents had received ≤8years of education (shown in Table 1).

**Table 1:** Demographic characteristics of the study population (*N*=1,317).

Variables	*n*	Percentage (%)
**Gender**		
Women	779	59.1
Men	538	40.9
**Grade**		
Freshman	687	52.2
Sophomore	364	27.6
Junior	134	10.2
Senior students	132	10.0
**Subject category**		
Liberal arts	460	34.9
Sciences	857	65.1
**Father’s educational level**		
Junior high school or below	945	71.8
High school	215	16.3
Junior college or college	155	11.8
Master or above	2	0.15
**Mother’s educational level**		
Junior high school or below	1,081	82.1
High school	155	11.8
Junior college or college	78	5.9
Master or above	3	0.23

### Measurements

#### Family Support of e-Learning

The Students’ Perception of E-learning Family Support Questionnaire (SPEFSQ) is a three-dimensional and 14-item instrument that measures student perception of family support in their studies and life (Khallad and Jabr, 2016; Guo et al., 2020). It has three dimensions, including environmental support (six items, such as “my parents can provide a quiet environment for my online classes”), emotional support (four items, such as “my parents will fully respect my study arrangements and plans”), and capability support (four items, such as “when I encounter learning problems, my parents can find answers or solutions together with me in time”). Every item is calculated using a 7-point scale ranging from 7 to 1. All subjects can choose the most suitable statement according to their actual situation. The highest score of 7 was “in full agreement,” and the lowest score of 1 was “not relevant to me at all.” The total score of this questionnaire is the sum of the scores of each item, and higher scores reflect that families give students more concern and support in learning. The SPEFSQ was compiled by the research team according to their knowledge in combination with literature research, expert consultation and investigations of survey tools that showed satisfactory reliability and validity among college students. In our samples, Cronbach’s alpha of the entire questionnaire was 0.952, and for the three subscales, it was 0.934 (environmental support), 0.890 (emotional support), and 0.902 (capability support), and KMO was 0.941. The SPEFSQ had been shown to three-factor structure: environmental support [average variance extracted (AVE)=0.736, composite reliability (CR)=0.944, and discriminant validity (DV)=0.858], emotional support (AVE=0.616, CR=0.863, and DV=0.785), and capability support (AVE=0.656, CR=0.884, and DV=0.810).

#### Student e-Learning Engagement

Student e-learning engagement was assessed by using a reliable and validated instrument – the Students’ E-learning Engagement Scale (SEES). Because our research object is web-based learners, Schaufeli’s original SEES scale was revised on the basis of literature research, expert consultation, and a pretest (Ouweneel et al., 2011; Siang and Santoso, 2016). The scale has 3 dimensions and 16 items, including absorption, dedication, and vigor. Student responses were scored on a 7-point Likert scale ranging from 7 (in full agreement) to 1 (not relevant to me at all). In the whole statistical analysis, the mean of the total scale score was used, and higher scale scores indicate higher student learning engagement. In our samples, Cronbach’s alpha of the SEES was 0.970, and for the three subscales, it was 0.926 (absorption), 0.902 (dedication), and 0.946 (vigor), and KMO was 0.965. The SEES had been shown to three-factor structure: absorption (AVE=0.657, CR=0.920, and DV=0.811), dedication (AVE=0.682, CR=0.895, DV=0.826), and vigor (AVE=0.729, CR=0.942, and DV=0.854).

#### Student e-Learning Self-Efficacy

The Student E-learning Self-efficacy (SES) scale was developed by Pintrich and De Groot (1990) and was revised by Yusong (2000). It consists of 2 dimensions and 22 items. The first dimension of the scale assesses learning capability self-efficacy, and the second dimension assesses learning behavior self-efficacy. The total scale score was the sum of these two dimensions, where higher scale scores indicate the higher learning self-efficacy of college students. The scale scoring system used a 7-point Likert scale ranging from 7 (in full agreement) to 1 (not relevant to me at all). In our samples, Cronbach’s alpha of the student e-learning self-efficacy scale was 0.919, and for the two subscales, it was 0.906 (learning capability self-efficacy) and 0.876 (learning behavior self-efficacy), and KMO was 0.933. The SES had been shown to two-factor structure: learning capability self-efficacy (AVE=0.739, CR=0.944, and DV=0.860) and learning behavior self-efficacy (AVE=0.548, CR=0.934, and DV=0.740).

#### Student e-Learning Normative Consciousness and Behaviors

Student e-learning normative consciousness and behaviors were evaluated using six items. Item 1: “I attend classes on time every time”; Item 2: “Before and after class, I will preview or review”; Item 3: “In class, I can follow the teacher’s ideas to learn”; Item 4: “In class, I will think deeply about the teacher’s questions”; Item 5: “In class, I will listen to other students’ answers or reports to enrich my knowledge or understand different views”; and Item 6: “After class, I can finish my homework on time.” Student responses were scored on a 7-point Likert scale ranging from 7 (in full agreement) to 1 (not relevant to me at all). All subjects could choose the most suitable statement according to their actual situation. In our samples, Cronbach’s alpha of the student e-learning normative consciousness and behaviors scale was 0.911 and KMO was 0.886.

### Statistical Analysis

All data were managed and analyzed using Statistical Package for the Social Sciences software (SPSS, version 26.0) and Excel (Microsoft Corp, Redmond, WA, United States). Descriptive statistics were applied to analyze the demographic data and all study variables. The correlations between study variables were analyzed by Pearson’s correlation, and the degree of these correlations was divided into three levels: small (correlation coefficient approximately 0.10), medium (correlation coefficient near 0.30), and large (for correlation coefficient ≥0.50; Cohen, 1988). The mediation model was tested with PROCESS Model 6 using 5000 bootstrap samples in SPSS. The bias-corrected bootstrap method can provide the highest statistical efficacy and the most accurate confidence interval estimation (Fang et al., 2012). Family support was used as an independent variable, e-learning normative consciousness and behaviors and e-learning self-efficacy were used as mediating variables, and student e-learning engagement was used as a dependent variable. The total, direct, and indirect effects were considered statistically significant at the 0.05 probability level if the results of the 95% bias-corrected confidence interval (CI) did not include zero (Hayes, 2013).

### Ethical Approval

The study protocol was approved by the ethics committees of University of South China.

## Results

### Pearson Correlation Analysis Results

Table 2 shows the overall Pearson correlation results. All study variables were significantly correlated with each other. Student e-learning engagement was positively and strongly correlated with family support (*r*=0.475, *p*<0.001), e-learning normative consciousness and behaviors (*r*=0.707, *p*<0.001), and e-learning self-efficacy (*r*=0.724, *p*<0.001).

**Table 2:** Pearson correlations among study variables.

Variables	1	2a	2b	2c	3a	3b	4a	4b	4c
1. E-learning normative consciousness and behaviors	1								
2. Student e-learning engagement									
a. Absorption	0.665^**^	1							
b. Dedication	0.683^**^	0.809^**^	1						
c. Vigor	0.673^**^	0.801^**^	0.814^**^	1					
3. E-learning self-efficacy									
a. Learning behavior self-efficacy	0.530^**^	0.604^**^	0.625^**^	0.632^**^	1				
b. Learning capability self-efficacy	0.624^**^	0.679^**^	0.675^**^	0.692^**^	0.741^**^	1			
4. Family support									
a. Capability support	0.437^**^	0.413^**^	0.442^**^	0.446^**^	0.495^**^	0.544^**^	1		
b. Emotional support	0.481^**^	0.415^**^	0.446^**^	0.431^**^	0.452^**^	0.522^**^	0.724^**^	1	
c. Environmental support	0.472^**^	0.380^**^	0.410^**^	0.377^**^	0.416^**^	0.499^**^	0.665^**^	0.798^**^	1
*M*	32.929	27.648	18.995	27.533	57.949	30.543	21.544	23.090	35.820
*SD*	5.998	6.546	4.596	6.953	10.836	6.887	5.549	4.862	6.850

*N*=1,317.

** All correlations were significant at *p*<0.001 (two tailed).

### Mediation Effect Models of Study Variables

**Figure 1:**
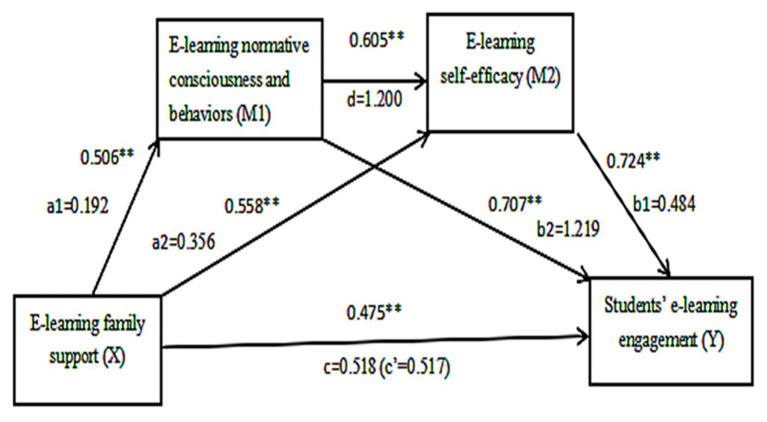
Mediation model of how family support influences students’ e-learning engagement *via* e-learning normative consciousness and behaviors and e-learning self-efficacy. All study variables are significantly correlated with each other. ^**^p<0.001. a1, direct effect of X on mediator M1; a2, direct effect of X on mediator M2; b1, direct effect of mediator M2 on Y; b2, direct effect of mediator M1 on Y; d, direct effect of mediator M1 on mediator M2; c, total effect of X on Y; and c’, direct effect of X on Y.

E-learning normative consciousness and behaviors and e-learning self-efficacy were identified as mediators between family support and student e-learning engagement. Furthermore, we found that there was not multicollinearity among those variables (Tol>0.1, VIF<10; shown in Table 3). In the SPSS PROCESS tool, model 6 was used to analyze the mediation effect of the study variables. The direct effect and indirect effect results are summarized in Tables 4 and 5. Figure 1 shows our research mediation effect model. The total effect of the model was statistically significant (*B*=0.517, *t*=19.567, *p*<0.001). The direct effect of family support on student learning engagement was not significant (*B*=−0.001, *t*=−0.048, *p*=0.962). The indirect effect of family support (X) on student e-learning engagement (Y) through e-learning normative consciousness and behaviors (M1) was significant, *B*=0.234, *SE*=0.021, 95% CI (0.195, 0.277). The mediation effect (X→M1→Y) accounted for 45.26% of the total effect. Additionally, e-learning self-efficacy (M2) mediated the relationship between family support and student e-learning engagement, *B*=0.172, *SE*=0.017, 95% CI (0.141, 0.207). The mediation effect (X→M2→Y) accounted for 33.27% of the total effect. Finally, the indirect effect of family support (X) on student e-learning engagement (Y) through e-learning normative consciousness and behaviors (M1) and e-learning self-efficacy (M2) was also found, *B*=0.112, *SE*=0.015, 95% CI (0.084, 0.143). The mediation effect (X→M1→M2→Y) accounted for 21.66% of the total effect. Therefore, the results of the mediation indicated that e-learning normative consciousness and behaviors (M1) and e-learning self-efficacy (M2) fully mediate the influence of family support on student e-learning engagement.

**Table 3:** Collinearity diagnosis among variables in the model.

Model		Collinearity statistics	
		Tolerance	VIF
1.	E-learning normative consciousness and behaviors	0.593	1.687
2.	Family support	0.644	1.553
3.	E-learning self-efficacy	0.549	1.822

**Table 4:** Direct effect results of mediation analysis.

Dependent variable	Independent variable	coeff	se	*t*	*p*	LLCI	ULCI
E-learning normative consciousness and behaviors (M1)	constant	17.463	0.740	23.586	<0.001	16.011	18.916
	Family support (X)	0.192	0.009	21.287	<0.001	0.175	0.210
E-learning self-efficacy (M2)	constant	20.372	2.100	9.699	<0.001	16.251	24.492
	M1	1.200	0.066	18.296	<0.001	1.071	1.329
	Family support (X)	0.356	0.025	14.280	<0.001	0.307	0.404
Student e-learning engagement (Y)	constant	−8.759	1.831	−4.784	<0.001	−12.350	−5.167
	M1	1.219	0.062	19.713	<0.001	1.098	1.341
	M2	0.484	0.023	20.859	<0.001	0.439	0.530
	Family support (X)	−0.001	0.023	−0.048	0.962	−0.045	0.043
Student e-learning engagement (Y)	constant	32.554	2.168	15.018	<0.001	28.302	36.807
	Family support (X)	0.517	0.026	19.567	<0.001	0.465	0.569

**Table 5:** Indirect effect results of mediation analysis.

	Effect	BootSE	BootLLCI	BootULCI
Total	0.518	0.029	0.465	0.578
Ind1 (X-> M1-> Y)	0.234	0.021	0.195	0.277
Ind2 (X-> M1-> M2-> Y)	0.112	0.015	0.084	0.143
Ind3 (X-> M2-> Y)	0.172	0.017	0.141	0.207
C1 (Ind1-Ind2)	0.123	0.027	0.072	0.178
C2 (Ind1-Ind3)	0.062	0.028	0.008	0.118
C3 (Ind2-Ind3)	−0.061	0.023	−0.103	−0.016

Because the three indirect effects (including X→M1→Y, X→M2→Y, and X→M1→M2→Y) were statistically significant, we tested whether these effects were significantly different in the mediation effects. We found that the mediating effect of e-learning normative consciousness and behaviors was the strongest.

## Discussion

The results of our study confirmed the research hypothesis. The mediating effects of student e-learning normative consciousness and behaviors and self-efficacy may contribute to understanding the relationship between family support of e-learning and e-learning engagement in college students. In the mediator model, a full intermediary effect existed.

### Direct Relations

When analyzing direct effects in this study, family support of e-learning positively predicted student e-learning engagement. This indicates that students who receive more interest and help from family members tend to have a high level of learning engagement. Our findings concurred with prior studies of the relationship between family support and learning engagement (Garciareid et al., 2015; Mazurenko and Hearld, 2015; Frawley et al., 2019). Due to the COVID-19 pandemic, universities, middle schools, and primary schools in many countries have launched online teaching and learning using the Internet. Most of the courses have changed from traditional face-to-face teaching to online teaching. With the change in teaching methods, educators and parents are most concerned about how much students have learned and whether they have listened carefully. To facilitate the process of online learning, family support has a great impact on e-learning engagement. Family support covers environmental support, emotional support, and capability support. Concerning environmental support, consistent with some studies (Elliot et al., 2017; Ericsson et al., 2018), a healthy and harmonious family environment can strengthen student learning engagement. In addition, studies indicate that family support may promote students’ positive or negative emotional experiences with learning (Nalavany and Carawan, 2012; Carawan et al., 2016; Frawley et al., 2019). Long-term emotional depression is negatively related to student learning ability and educational success (Nalavany and Carawan, 2012). Finally, family capability support plays an integral role in e-learning engagement, for example, whether parents can participate in solving learning difficulties (purchase and maintain online learning equipment, provide an unobstructed network, use new learning software, etc.) and whether they can give constructive suggestions according to the actual learning situations. Taken together, family support, as a form of social support, can contribute to the development of learning competencies and learning motivation and enhance student e-learning engagement.

### Mediated Relations

As hypothesized, we found that the influence of family support on student e-learning engagement was fully mediated by e-learning normative consciousness and behaviors and self-efficacy: Students who perceived that their family members supported their e-learning experienced high levels of learning engagement because they thought they could consciously abide by the norms of learning behavior and felt they had the capacity to devote themselves to learning.

In accordance with social cognitive theory (Bandura, 1997) and self-efficacy theory (Jiang et al., 2019; Codella et al., 2020; Li et al., 2020), we confirmed a mediating role of self-efficacy in the association between family support and student e-learning engagement, which means that promoting family support as a way to improve students’ sense of self-efficacy could help students put more energy into e-learning. As an important variable, self-efficacy can influence motivation and learning in student activities and play a decisive role in learner behavior by affecting various personal dimensions, such as focus, dedication, vigor, aspirations, and expectations (Codella et al., 2020; Wu et al., 2020). Therefore, the higher the learning self-efficacy of students, the more they will be involved in learning.

According to the mediation model in our study, the associations between family support, e-learning normative consciousness and behaviors, self-efficacy, and e-learning engagement can be explained. Learning normative consciousness and behaviors can mediate the associations between family support and e-learning engagement. Our result is consistent with self-regulated learning theory and metacognitive learning theory (Aurah, 2013; Şen, 2016; Ganda and Boruchovitch, 2018). In an unsupervised online learning environment, learning normative consciousness and behaviors play a fundamental role in high learning engagement. From the perspective of metacognitive learning theory, self-efficacy is one of the key determinants for learners to use among metacognitive learning strategies, such as the e-learning normative consciousness and behaviors in this study (Hayat et al., 2020). Our results also demonstrated that self-efficacy and learning normative consciousness and behaviors are closely related. Family members can encourage children and adolescents to form good learning normative consciousness and behaviors by establishing good quality parent-child interactions and relationships. Therefore, family member support both directly and indirectly enhanced student learning engagement by influencing learning normative consciousness and behaviors and self-efficacy.

### Implications

On the basis of previous studies, we further investigated the concurrent and systematic effects of the three variables of family support, learning normative consciousness and behaviors and self-efficacy on student e-learning engagement in a network-based learning context. The path, family support→learning normative consciousness and behaviors→self-efficacy→e-learning engagement, revealed the multiple mediating roles of learning normative consciousness and behaviors and self-efficacy in the relationship between family support and e-learning engagement. The influences of family support on e-learning engagement were statistically significant and fully mediated by e-learning normative consciousness and behaviors and self-efficacy. These results were the best interpretation of self-regulated learning theory and metacognitive learning theory (Aurah, 2013; Şen, 2016; Ganda and Boruchovitch, 2018) and reflected the specific guidance of these two theories to teaching practice. Moreover, these findings serve as practical guidelines for parents and teachers to consciously and intentionally create an effective learning environment and cultivate student learning normative consciousness and behaviors to foster their engagement in e-learning. To go a step further, family members can increase family support of their children by creating a household environment conducive to learning, displaying positive emotions, demonstrating the capability to assist their children, advocating the significance of learning normative consciousness and behaviors, and encouraging dedicated and efficient learning. In this regard, students will develop more learning normative behaviors and self-efficacy and subsequently engage more in e-learning.

So far, the irreplaceable of e-learning is more and more obvious. Its advantages determine its popularity, but its disadvantages highlight the importance of this study. E-learning based on network challenges people’s self-discipline almost every moment, which will directly affect learning investment and learning efficiency (Poon et al., 2015). Good e-learning normative consciousness and behaviors are the embodiment of one’s self-discipline. Its formation needs a long process, which is different from the traditional face-to-face classroom learning. Harmonious family environment, long-term emotional support, and capability support among family members can contribute to the formation of good e-learning normative consciousness and behaviors, which will greatly improve students’ e-learning engagement. Moreover, once this kind of normative consciousness and behavior is formed, its effect will be stable and lasting. On a deeper level, learning engagement can maintain a good level for a long time. This provides a feasible and effective way to improve students’ learning engagement.

### Strengths, Limitations, and Future Study

The strength of our study first analyzed the effects of e-learning normative consciousness and behaviors and self-efficacy on the relationship between family support and e-learning engagement in college students. These findings will provide a new way to effectively intervene students’ learning engagement, especially in the network learning environment. These data may provide a reference for students, students’ parents, teachers, and education administrators in pursuit of high efficiency of students’ autonomous learning.

As with all research, this study has several limitations. First, we found that reciprocal relationships could exist between the variables, but cross-sectional data cannot draw causal conclusions about these relationships. Second, at present, our research has only been carried out in colleges and universities, and science students are in the majority; it is not clear whether this model will be mediated by other factors in primary and secondary schools. Finally, this study is mainly carried out in the e-learning environment based on Internet, and the effect in traditional teaching needs to be further verified. Further research possibilities include the following: seek the best intervention model of learning engagement for different education groups and study the intervention costs of learning engagement to obtain the maximum benefit with the minimum cost.

## Data Availability Statement

The raw data supporting the conclusions of this article will be made available by the authors, without undue reservation.

## Ethics Statement

The study protocol was approved by the ethics committees of University of South China. Written informed consent to participate in this study was provided by all subjects.

## Author Contributions

HG, YO, and LL formulated the study questions and directed their implementation. HG, YO, ZZ, MN, XZ, and LL participated in the data collection. HG and LL drafted the article. All authors were involved in revising the article and have approved this final version.

### Conflict of Interest

The authors declare that the research was conducted in the absence of any commercial or financial relationships that could be construed as a potential conflict of interest.
